# Simulation-based learning combined with debriefing: trainers satisfaction with a new approach to training the trainers to teach neonatal resuscitation

**DOI:** 10.1186/1756-0500-6-251

**Published:** 2013-07-04

**Authors:** Harish J Amin, Khalid Aziz, Louis P Halamek, Tanya N Beran

**Affiliations:** 1 Department of Pediatrics, University of Calgary, C4-615, Alberta Children’s Hospital, 2888 Shaganappi Trail N.W., Calgary, Alberta T3B 6A8, Canada; 2 Medical Director, Royal Alexandra Hospital NICU, Neonatal Administration Room 5027 DTC, 10240 Kingsway, Edmonton, Alberta T5H 3V9, Canada; 3 Division of Neonatal and Developmental Medicine, Department of Pediatrics, Stanford University, Suite 315, 750 Welch Road, Palo Alto, CA 94305, USA; 4 Community Health Sciences, University of Calgary, Calgary, Alberta T2N 4N1, Canada

**Keywords:** Neonatal resuscitation, Simulation, Debriefing

## Abstract

**Background:**

Prompt initiation of appropriate neonatal resuscitation skills is critical for the neonate experiencing difficulty transitioning to extra-uterine life. The use of simulation training is considered to be an indispensable tool to address these challenges. Research has yet to examine the effectiveness of simulation and debriefing for preparation of trainers to train others on the use of simulation and debriefing for neonatal resuscitation. This study determines the degree to which experienced NRP instructors or instructor trainers perceived simulation in combination with debriefing to be effective in preparing them to teach simulation to other health care professionals.

**Methods:**

Participants’ perceptions of knowledge, skills, and confidence gained following a neonatal resuscitation workshop (lectures; scenario development and enactment; video recording and playback; and debriefing) were determined using a pre-post test questionnaire design. Questionnaire scores were subjected to factor and reliability analyses as well as pre- and post-test comparisons.

**Results:**

A total of 17 participants completed 2 questionnaires. Principal component extraction of 18 items on the pre-test questionnaire resulted in 5 factors: teamwork, ability to run a simulation, skills for simulation, recognizing cues for simulation and ability to debrief. Both questionnaire scores showed good reliability (α: 0.83 - 0.97) and factorial validity. Pre- and post-test comparisons showed significant improvements in participants’ perceptions of their ability to: conduct (as an instructor) a simulation (p < .05, η^2^ .47); participate in a simulation (p < .05, η^2^ .45); recognize cues (p < .05, η^2^ .35); and debrief (p < .05, η^2^ .41).

**Conclusions:**

Simulation training increased participants’ perceptions of their knowledge, skills, and confidence to train others in neonatal resuscitation.

## Background

Concerns related to errors in healthcare practice have led to the call for more education and training in patient safety [1]. The science underlying neonatal resuscitation is growing exponentially in quality and quantity. Prompt initiation of appropriate neonatal resuscitation skills is critical for the neonate experiencing difficulty transitioning to extra-uterine life. The use of simulation training is considered to be an indispensable tool to address these challenges [2]. Its re-creation of characteristics of real clinical encounters provides opportunities for learning in various areas of health care. Utilization in the Neonatal Resuscitation Program (NRP) [3,4] indicates some positive impact on learner outcomes [5-8]. The addition of team training to the NRP curriculum results in improved communication and a marked reduction in time required to complete a simulated megacode [9]. In combination with debriefing, which is a semi-structured conversation about errors and strategies for individual and team improvement [10], there is some evidence of enhanced practice [11,12]. Research has yet to examine the effectiveness of simulation and debriefing for preparation of trainers to train others on the use of simulation and debriefing for neonatal resuscitation. The objective of our pilot study was to determine the degree to which experienced NRP instructors or instructor trainers perceived simulation in combination with debriefing to be effective in preparing them to teach simulation to other health care professionals.

## Methods

A total of 17 individuals (5 physicians, 4 clinical nurse educators, 3 nurse practitioners, 2 respiratory therapists, and 3 administrators) from Alberta, Canada; who were experienced NRP instructors, were purposely selected for the training. The individuals were experienced interprofessional NRP instructors or instructor trainers from a wide geographical area (Province of Alberta, Canada).

The facilitator was an expert trainer in simulation, debriefing and teamwork.

In order to adopt simulation, briefing and debriefing techniques in an effective education program for acquisition and maintenance of skills necessary for effective neonatal resuscitation, a 2-day workshop was designed that included: lectures; scenario development and enactment; video recording and playback; and debriefing. The objective was to enhance the capacity of experienced NRP instructors by shifting their roles from that of a teacher responsible for imparting knowledge to trainees to that of a facilitator who fosters acquisition of skills by trainees as they accept primary responsibility for their own training. The instructors would learn the knowledge, skills and behaviors required to: a) facilitate simulation-based learning by giving learners key visual, auditory and tactile cues (e.g., baby’s cry, expiratory grunting, reduced tone) so that they can display authentic skills; b) observe learner interactions with their teams and environments; and c) debrief those experiences.

Day 1 of the training included: i) review of the evolution of NRP in the United States and discussion of current neonatal resuscitation training in Canada; ii) an overview of simulation-based learning in neonatal resuscitation; iii) a discussion of designing and conducting realistic, challenging scenarios on a realistic, challenging budget; and iv) an interactive session on debriefing. Day 2 comprised of: running clinical scenarios; conducting debriefing; and then discussing the debriefing. Debriefing focused on creating a supportive learning environment; and understanding roles and responsibilities of all team members. The interactive learning included: leading a debriefing; generating a debriefing checklist based on learning objectives; standardizing responses of simulator instructors; and defining the role of the instructor in the debriefing process. All participants attended all sessions, and scenarios were captured on time-coded videotape for playback, which were discussed during the debriefing. This discussion included: a review of decisions made; actions that could have been performed differently in the scenario; and tasks that were successfully accomplished. An expert provided the experienced NRP instructors feedback on the effectiveness of their debriefing.

The Neonatal Resuscitation Simulation Self-Assessment Questionnaire (Appendix 1) consists of 18 items that measure respondents’ perceptions about the degree of knowledge, skills, and confidence they have in several areas of neonatal resuscitation simulation that were addressed in the training. These areas include: developing standardized scenarios; recognizing proficient behavioral skills such as teamwork; performing appropriate technical skills; conducting effective debriefings; and using patient simulator and audiovisual devices. Responses to all of the items were provided along a 5-point scale ranging from “strongly agree” to “strongly disagree”. This questionnaire was administered before and after participants completed the training to determine if they perceived changes in their abilities to conduct simulation.

The Neonatal Resuscitation Simulation Appraisal Questionnaire (Appendix 2) was administered after the training to obtain participants’ suggestions for improvement. It contains 13 questions that were developed based on input from content experts in neonatal resuscitation and simulation for content validity. The first section lists three open-ended questions that ask about important areas addressed in the training, what worked well, and ideas for improvement. The second section consists of 7 questions about the overall value of the training and the specific areas that were covered. These responses were organized along a 5-point response scale from “strongly agree” to “strongly disagree”. An additional question asked about the extent to which participant expectations were met. Respondents answered “not at all met”, “partially met”, or “completely met”. The internal consistency of these items according to Cronbach’s alpha was .75, indicating good reliability. In the third section two questions asked participants to rate the presenter on a 5-point scale from “excellent” to “very poor”, and to rate the length of the training on a 3-point scale from “too long” to “too short”.

Both these questionnaires were designed in-house for study purposes.

## Ethics

The Conjoint Health Research Ethics Board of the University of Calgary approved this study.

## Results

Using pre-test data only, the 18 items on the Neonatal Resuscitation Simulation Self-Assessment Questionnaire were subjected to principal component extraction, resulting in five factors. The total explained variance was 87.02%, and the rotation converged in 7 iterations. The reliability of the items measured within each of the factors was high (Cronbach’s alphas ranged from .83 to .97). Using the means of the items that loaded under each of the five factors, we determined that four of them significantly increased after the training. Perceptions of teamwork did not significantly increase, *t*(28) = 1.64, *p* = .11. However, perceptions of the ability to use equipment in a simulation increased from pre- (*M* = 3.06, SD = 0.59) to post-test (*M* = 4.02, SD = 0.44), *t*(27) = 4.86, *p* = .000, partial η^2^ **=** .47. Perceived technical skills improved from pre- (*M* = 2.81, SD = 0.88) to post-test (*M* = 4.07, SD = 0.49), *t*(28) = 4.75, *p* = .000, partial η^2^ **=** .45. Perceived familiarity with cues increased from pre- (*M* = 3.29, SD = 0.74) to post-test (*M* = 4.19, SD = 0.50), *t*(28) = 3.84, *p* < .001, partial η^2^ **=** .35, and perceived ability to debrief increased from pre (*M* = 2.75, SD = 0.70) to post-test (*M* = 3.86, SD = 0.66), *t*(28) = 4.41, *p* = .000, partial η^2^ **=** .41.

Comments about several aspects of the training are presented in Table 1. The distribution of responses suggests that the majority of participants considered the training to be valuable and learned how to: develop scenarios; use high and low fidelity equipment; provide cues; appreciate the importance of teamwork; debrief; and develop technical skills. Relatively few participants provided a neutral rating, and only one participant disagreed with one aspect of the training.

**Table 1:** **Number (%) of participants evaluating each aspect of the training (*N*** **= 13, 4 missing)**

	**Strongly agree**	**Agree**	**Neutral**	**Disagree**	**Strongly disagree**
Training valuable	7 (53.8)	6 (46.2)	0	0	0
Develop scenarios	5 (38.5)	6 (46.2)	2 (15.3)	0	0
Use low/hi fidelity equipment	2 (15.3)	8 (61.5)	3 (23.2)	0	0
Provide cues	4 (30.8)	8 (61.5)	1 (7.7)	0	0
Importance of teamwork	7 (53.8)	6 (46.2)	0	0	0
Debriefing	7 (53.8)	6 (46.2)	0	0	0
Develop technical skills	2 (15.3)	5 (38.5)	5 (38.5)	1 (7.7)	0

In addition, 61.5% (*n* = 8) of respondents rated the presenter as very good, and 38.5% (*n* = 5) rated him as excellent. Also, the majority of respondents (76.9%, *n* = 10) reported that the training was a good length, and three respondents (23.1%) stated that it was too short.

To obtain additional comments, we conducted qualitative analyses of the responses to the three open-ended questions on the Neonatal Resuscitation Simulation Appraisal Questionnaire. The content analysis method, as presented in Berg [13], when applied to responses about the most important issue addressed in the training yielded four different themes. Of the 12 respondents who completed this question, half of them (50.0%) indicated that learning debriefing methods was critical. A quarter (25.0%) indicated that learning how to conduct simulation was important. Some respondents reported that addressing the learners’ needs (16.7%) and having an opportunity to apply their learning (16.7%) were also important issues addressed (one respondent provided two answers). When asked about aspects that worked well, 45.2% indicated debriefing. Also, 30.8% indicated practicing simulation, and another 30.8% of respondents indicated using the scenarios (four respondents provided two answers). On the third question, 11 respondents provided suggestions for improvement. Over a third (36.4%) stated the scenarios could have been more relevant, and another third (36.4%) indicated there were some teaching problems (e.g., too much lecture, technical problems). Another 27.2% stated the training was too short and requested more time and practice. The inter-rater reliability between two judges for the three questions ranges from .84 to .91, with a mean of .88, indicating good consistency in scoring.

## Discussion

This study showed that trainers perceive simulation in combination with debriefing to be effective in preparing them to teach simulation to other health care professionals. The majority of participants thought that the training was well facilitated and valuable for improving knowledge, skills, and confidence in neonatal resuscitation simulation training. They also rated highly the importance of debriefing, and provided important suggestions about developing relevant scenarios, reducing lecture, and increasing time and practice to improve the training in the future.

Our finding that half of the participants rated debriefing as the most effective aspect of this training is congruent with a considerable body of evidence in the education literature that debriefing “… can have a very powerful effect on learning” [14]. Indeed, studies have shown that feedback through dialogue between the teacher and learner promotes learning [15].

Conducting a simulation learning session ranked second in importance, and addressing the learners’ needs and opportunities to apply their learning were also reported as important factors for learning. These responses reflect the need for adult learners to gain practical experience. Lack of such experience may be seen as a barrier to introducing simulation into a resuscitation curriculum.

Participants reported significant improvement in their knowledge, skills, and confidence in: using equipment in a simulation; developing technical skills; gaining familiarity with cues; and conducting debriefing. They did not, however, report improvement in teamwork, as measured by ratings of their ability to work as a team and address team members’ needs. This result stands in contrast to some research [9] that supports debriefing as valuable in improving team performance. Through examining the qualitative responses we found that over a third of participants found the scenarios could have been more representative of the types of clinical cases they encounter. Thus, it is possible that they found the scenarios used in the training did not provide realistic examples of cases relevant to team members, which would impair their ability to work cooperatively together and make decisions towards common goals [16]. We recommend that for these types of training, participants be given the opportunity to provide input into the scenarios they would like to practice in the sessions. In this way, the scenarios will be perceived as a better match to their own learning needs and experiences, and they directly participate in the development of realistic scenarios to increase their ability to do so cooperatively in the future.

This study possesses a number of limitations. We were unable to request names of or identification numbers for respondents, and, thus, could not match responses between pre- and post-test: this reduces the probability of finding significant changes between the two time intervals when the questionnaires were administered. We therefore cannot provide prospective data on the performance of the participants in clinical practice. Notwithstanding, we did find that four of the five areas significantly improved. As comments from 4/17 (24%) individuals are missing and because participants were specifically selected , these results may not be generalizable. Despite having demanding schedules, many participants stated that two days was not a sufficient allocation of time. It is recommended that this type of training focus more on debriefing and management of scenarios with less time devoted to lectures and review of general simulation information. Given the positive perceptions gained in this pilot study, evidence of gains in knowledge and skills to teach neonatal resuscitation with simulation will be examined next in an evaluation study.

## Conclusions

Simulation training increased participants’ perceptions of their knowledge, skills, and confidence to train others in neonatal resuscitation. Clinical experience is not a proxy for simulation instructor effectiveness. Feedback and debriefing have an essential role in simulation-based medical education. Trainers perceive simulation in combination with debriefing to be effective in preparing them to teach simulation to other health care professionals. Learning how to debrief following an instruction session is critical for instructor effectiveness.

## Appendix 1

### Neonatal resuscitation simulation self-assessment questionnaire

1. I have the knowledge to develop standardized scenarios that are challenging and appropriate for the level of learner and provider. Strongly agree Agree Neutral Disagree Strongly disagree

2. I have skills to develop standardized scenarios that are challenging and appropriate for the level of learner and provider. Strongly agree Agree Neutral Disagree Strongly disagree

3. I am confident in developing standardized scenarios that are challenging and appropriate for the level of learner and provider. Strongly agree Agree Neutral Disagree Strongly disagree

4. I have the knowledge to use low and high fidelity equipment. Strongly agree Agree Neutral Disagree Strongly disagree

5. I have the skills to use low and high fidelity equipment. Strongly agree Agree Neutral Disagree Strongly disagree

6. I am confident in using low and high fidelity equipment. Strongly agree Agree Neutral Disagree Strongly disagree

7. I have the knowledge to provide visual, auditory and tactile cues for simulation. Strongly agree Agree Neutral Disagree Strongly disagree

8. I have the skills to provide visual, auditory and tactile cues for simulation. Strongly agree Agree Neutral Disagree Strongly disagree

9. I am confident in providing visual, auditory and tactile cues for simulation. Strongly agree Agree Neutral Disagree Strongly disagree

10. I have knowledge regarding effective teamwork in a resuscitation setting. Strongly agree Agree Neutral Disagree Strongly disagree

11. I have the skills necessary to work as a member of a team in a resuscitation setting. Strongly agree Agree Neutral Disagree Strongly disagree

12. I have confidence in my ability to work as a member of a team in a resuscitation setting. Strongly agree Agree Neutral Disagree Strongly disagree

13. I have knowledge regarding the art of debriefing. Strongly agree Agree Neutral Disagree Strongly disagree

14. I have skills in the art of debriefing. Strongly agree Agree Neutral Disagree Strongly disagree

15. I have confidence in my ability to facilitate a debriefing. Strongly agree Agree Neutral Disagree Strongly disagree

16. I have knowledge regarding the technical skills used in simulation. Strongly agree Agree Neutral Disagree Strongly disagree

17. I have technical skills used in simulation. Strongly agree Agree Neutral Disagree Strongly disagree

18. I have confidence in my technical skills to facilitate a simulation. Strongly agree Agree Neutral Disagree Strongly disagree

## Appendix 2

### Neonatal resuscitation simulation appraisal questionnaire

We would be very grateful if you could provide feedback on the workshop.

1. What do you think was the most important issue that was addressed?

2. Please list aspects of the workshop that worked well.

3. How do you think the workshop could have been improved?

4. Overall, to what extent were your expectations met in the workshop? Not at all met Partially met Completely met

5. I found the workshop valuable: Strongly agree Agree Neutral Disagree Strongly disagree

6. I learned how to develop standardized scenarios that are challenging and appropriate for the level of learner and provider. Strongly agree Agree Neutral Disagree Strongly disagree

7. I learned how to use low and high fidelity equipment. Strongly agree Agree Neutral Disagree Strongly disagree

8. I learned how to provide visual, auditory and tactile cues for simulation. Strongly agree Agree Neutral Disagree Strongly disagree

9. I learned the importance of teamwork in a resuscitation setting. Strongly agree Agree Neutral Disagree Strongly disagree

10. I learned the art of debriefing. Strongly agree Agree Neutral Disagree Strongly disagree

11. I learned how to develop technical skills used in simulation. Strongly agree Agree Neutral Disagree Strongly disagree

12. I would rate expert facilitator’s presentation as: Excellent Very good O.K Poor Very Poor

13. I would rate the length of the workshop as: Too long Good length Too short

## Competing interests

Dr. Halamek is a consultant to Laerdal Medical, Inc., and Advanced Medical Simulations, Inc.; his effort is partially supported by the Endowment for the Center for Advanced Pediatric and Perinatal Education at Packard Children’s Hospital at Stanford. All other authors have no competing interests to declare.

## Authors’ contributions

HA and TB oversaw all aspects of the study, and drafted the manuscript. KA and LH were co-investigators who provided critical input into the manuscript. TB conducted the data analysis. All authors read and approved the final manuscript.
